# Effects of Light Level and Nitrogen Supply on the Red Clover–*Orobanche Minor* Host–Parasite Interaction

**DOI:** 10.3390/plants8060146

**Published:** 2019-05-31

**Authors:** Joel I. Jokinen, Louis J. Irving

**Affiliations:** School of Life and Environmental Sciences, University of Tsukuba, 1-1-1 Tennodai, Tsukuba 305-8577, Japan; joel.jokinen@hotmail.fi

**Keywords:** *Trifolium pratense*, *Orobanche minor*, parasitism, photosynthesis, nitrogen deficiency

## Abstract

Infection by holoparasitic plants typically causes decreases in host mass, thought to be primarily as a result of resource abstraction. Inverse relationships have been noted between the number of *Orobanche* spp. parasites infecting a host and their mass, suggesting that the parasites compete for a shared resource pool, assumed to be recently fixed carbon (C). In clover, nitrogen (N) fixation requires a high proportion of daily photosynthate and represents a potential competitor for recently fixed C. We grew *Trifolium pratense*, either singly or parasitised by *Orobanche minor*, under high or low light levels, and with or without exogenous N supply. Low light and N deficiency led to decreased host biomass, while the damage caused by parasitism was proportionate to host mass. Parasitism caused reductions in host leaf mass, area, photosynthetic rates and shoot N concentration, but did not affect starch accumulation. Parasite mass as a proportion of system biomass was significantly higher when attached to plants grown at high light, which was attributed to higher photoassimilate supply, while the N supply had no effect. While both N limitation and parasitism caused reductions in host growth, little evidence of competition for C between N fixation and the parasites was noted.

## 1. Introduction

Parasitic plants are traditionally split into hemiparasites and holoparasites. Hemiparasites are generally thought to be parasitic for nutrients and water, but fix some or most of their own carbon (C). Conversely, holoparasites are often non-photosynthetic, relying on their host as a C source. The holoparasite *Orobanche minor* derives all its nutritional needs from the host phloem via a specialised organ called the haustorium, with host–parasite phloem continuity between *Brassica napus* and the parasite *Orobanche ramosa* recently demonstrated using a fluorescent symplastic dye [1]. Other mechanisms of nutrient transfer from the host to the parasite, such as interfacial parenchyma cells, may also exist [2,3].

Holoparasite infection causes a decline in host growth. *Orobanche crenata* infection caused biomass reductions in faba bean, field pea and grass pea hosts, with an inverse relationship between individual parasite mass and the number of parasites per plant suggesting competition for a common resource pool, assumed to be recent photoassimilates [4]. Similarly, *Orobanche cernua* parasite attachment caused almost complete growth suppression of its tobacco host, with an exponential decline in the mass of individual *O. cernua* plants as the number of parasites per host plant increased [5]. However, the total system biomass did not markedly change as a result of parasite attachment, with host growth reductions resulting from mass transfer from the host to the parasite [5]. By contrast, in tomato plants (*Solanum lycopersicum*) parasitised by *Orobanche aegyptiaca*, the increase in parasite mass through the experiment was less than the decrease in host mass [6], leading to a decrease in system mass. In another study, although photosynthetic and nitrogen (N) uptake rates were respectively 21% and 23% higher in tobacco plants infected by *O. cernua* compared to unparasitised controls, nearly one-third of the total photosynthate and 27% of N taken up by the host was abstracted by the parasite, causing a net decrease in host C and N status [7]. 

In sunflower (*Helianthus annus*), C abstraction by *Orobanche cumama* has been shown to cause changes in host leaf morphology, similar to those exhibited by plants experiencing shade conditions [8]. Specific changes included increases in mean leaf area and the size of intercellular air spaces, and declines in mesophyll thickness and specific leaf mass, with these declines being greater in more strongly parasitised plants. While photosynthetic rates on a leaf area basis decreased, they increased on a whole-leaf basis. Taken together, this suggests a shift towards greater photosynthetic efficiency in host plants, with increased leaf areas for light harvesting, and decreased resistances to CO_2_ diffusion in host leaves. It was hypothesised that the host plants had become C depleted, leading both to changes in leaf morphology and to an increase in transpiration rates as stomatal aperture was increased to maximise photosynthesis [8]. 

N fixation in legumes is energetically costly, requiring approximately 2 mg C per mg N fixed in *Pisum sativum*, and 2.9 mg C per mg N in *Vivia faba* [9], and it has been estimated that N fixation accounts for nearly one-quarter of daily photosynthesis in white clover [10,11]. N fixation is understood to be primarily reliant on immediate photoassimilate supply, with long-term C stores being less important [12]. This implies the potential for C competition between the parasite and N fixation, with a 63% reduction in N fixation by *Lupinus albus*, resulting from infection by the shoot holoparasite *Cuscuta reflexa* [13], which was attributed to reduced C flux to the roots. Abbes et al. [14] measured the phloem sap concentrations of a variety of sugars, organic acids and amino acids in tolerant and susceptible faba bean varieties parasitised by *Orobanche foetida*, finding a general reduction in total carbohydrates in parasitised plants relative to controls. Phloem amino acid levels were reduced by 82% in the parasite-tolerant variety, but were unaffected in the susceptible variety, with lower soluble protein levels in *O. foetida* attached to the tolerant variety compared to the susceptible control. This suggests that the ability of the host plant to regulate phloem composition may be an important component in the host–parasite interaction. Similar reductions in xylem amino acid concentrations were noted in tobacco plants parasitised by *O. cernua* [7]. 

While recent work has shown that the xylem-feeding hemiparasite *Rhinanthus minor* cannot access N fixed by its legume host [15], the situation is less clear in phloem-feeding holoparasites like *O. minor*, with the assumption being that fixed N must cycle through the xylem to the phloem before being abstracted by the parasite [16]. However, rapid N transfer from a red clover (*Trifolium pratense*) host to *O. minor* without apparent cycling through the host shoot has been documented [17]. The parasite could abstract a majority of the ^13^NO_3_^−^ fed to parasitised roots, while abstraction from non-parasitised roots was negligible. These high transfer rates are interesting, given that nitrate is thought to be primarily transported via the xylem, and holoparasites such as *O. minor* are thought to derive relatively little nutrition from the xylem [7,16]. Phloem nitrate concentrations are generally considered to be low [18]. 

Much still remains unknown about the relationship between host plants and their parasites. Given the enormous economic and social impacts of plant parasitism, furthering our understanding of the mechanisms by which parasites cause damage upon their hosts is desirable. In the case of holoparasites, the primary mechanisms are generally considered to be the theft of C and other resources from the host plant, although this remains an understudied subject. *Orobanche minor* Sm. is a widely distributed invasive species in the Kanto region of eastern Japan. Its host, red clover (*Trifolium pratense* L.) is a widely used forage crop globally. As such, this pair represents a suitable model system for studies of the host–parasite interaction [17,19]. Given our understanding that holoparasites compete for plant resources, we hypothesised that shifts in host C–N balance would cause changes in the competitive balance in the three-way interaction between host growth, N fixation and the parasite. In order to test this, we grew *Trifolium pratense* plants either in the absence or presence of *Orobanche minor*, at control (High light; HL) or limiting light levels (Low light; LL) in the presence (High N; HN) or absence (Low N; LN) of an exogenous N supply. Photosynthetic rates are strongly dependent on light levels, and our low light conditions (80 µmol photons m^−2^ s^−1^) were chosen to cause a strong C limitation, compared to those grown under higher light conditions (180 µmol photons m^−2^ s^−1^), with N-deprived plants facing an additional C cost of N fixation. In the real world, light levels may be 1000 µmol photons m^−2^ s^−1^ or more, even on a cloudy day, under which conditions competition for C may be difficult to quantify. We hypothesised that (1) the growth of clover plants suffering C limitation as a result of low light and N deficiency would be more strongly affected by parasitism, (2) both high light and exogenous N supply would enhance parasite growth rates and (3) parasite attachment would have significant effects on host plant N and C status.

## 2. Results

### 2.1. Biomass Accumulation was Affected by Light, Nitrogen and Parasitism

**Figure 1:**
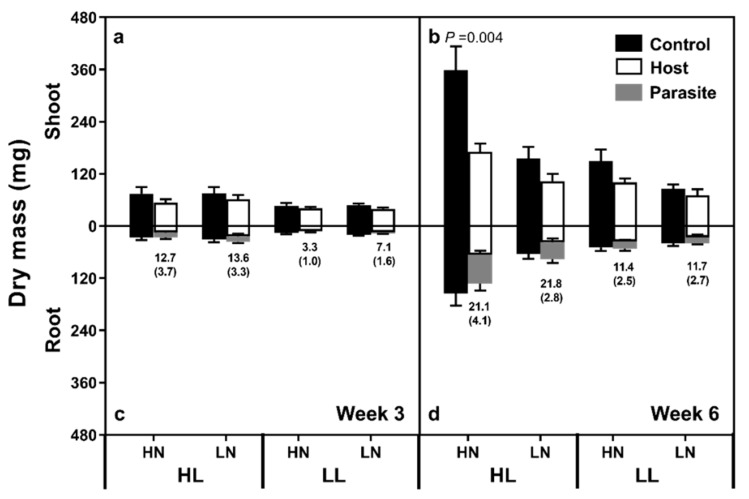
Clover host and parasite shoot (**a**,**b**) and root (**c**,**d**) mass after three (**a**,**c**) and six (**b**,**d**) weeks of treatments, where the bars represent standard errors, and the numbers represent the parasite as a percentage of total host mass. *p*-values denote significant differences between control and parasitised plants. HL and LL represent high light and low light treatments, while HN and LN represent high and low N treatments.

Three weeks after the start of treatments, the total system mass (host shoot + root + parasite) was 59% higher in HL than LL plants (Figure 1a, Table S1), but neither N supply nor parasitism had any effect. Host shoot mass was significantly higher in HL plants, while neither N level nor parasitism had an effect, and no interaction of effects was noted. Belowground biomass (host root + parasite) was significantly greater under HL than LL conditions, and under LN than HN conditions. Parasite mass was significantly greater in the HL than LL treatments (HL 11.6 mg ± 2.1; LL 2.7 mg ± 0.6; F_(1,20)_ = 12.058, *p* = 0.003), representing 37.2% (±5.1) and 17.0% (±3.5) of belowground biomass respectively, but N level had no effect nor were any effect interactions noted. 

After six weeks of treatment, the total system biomass (shoot + root + parasite) was significantly reduced in plants grown at LL and LN (Figure 1b). Total system biomass was reduced by parasitism, with 41% and 18% decreases noted in the HL/HN and HL/LN plants, and 23% and 12% declines noted in the LL/HN and LL/LN plants, respectively. However, no effect interactions were noted. Belowground biomass was significantly reduced under LL and LN conditions, while parasitism had no effect. Parasite mass was approximately 3.3 times higher in HL than LL treatments (HL 51.3 mg ± 9.0; LL 15.5 mg ± 2.4; F_(1,37)_ = 25.446, *p* < 0.001), representing 50.2% (±4.1) and 34.6% (±3.5) of belowground mass, respectively, with no effect of N level found.

### 2.2. Belowground: Aboveground Biomass was Higher in Parasitised Plants

**Figure 2:**
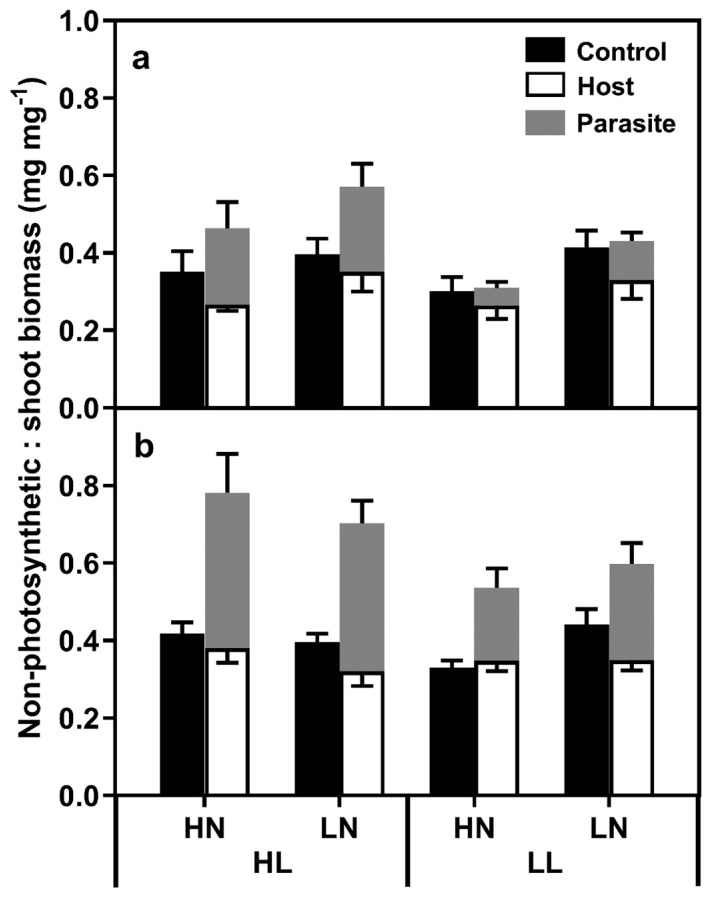
Host root and parasite mass per unit shoot mass (mg mg^−1^) after three (**a**) and six (**b**) weeks from the start of treatments, where the bars represent standard errors. HL and LL represent high light and low light treatments, while HN and LN represent high and low N treatments.

The mass of host roots and the parasites were divided by shoot mass to give a measure of differences in the balance between photosynthetic and non-photosynthetic tissues (Figure 2, Table S2). Considering only the host root, at week three, LN plants exhibited an increase in root mass per unit shoot mass (HN 0.297 mg mg^−1^ ± 0.020; LN 0.377 mg mg^−1^ ± 0.023), while parasitism caused a decrease (Control 0.366 mg mg^−1^ ± 0.022; Parasitised 0.302 mg mg^−1^ ± 0.021). At week six, parasitism caused a decrease in the root mass per unit shoots, and a light x N level interaction was noted, with simple effects analysis showing a significant difference between HN and LN root mass in control plants under LL conditions (F_(1,70)_ = 6.686, *p* = 0.012). For the parasite alone, significantly lower parasite mass per unit shoot material was noted under LL conditions at both weeks three and six, with no effects of N level or effect interactions noted. When the host root and parasite biomass was pooled, LL plants exhibited a relative decrease in belowground biomass at both weeks three and six. At week three, LN plants exhibited a higher relative biomass than HN plants (LN 0.455 mg mg^−1^ ± 0.029; HN 0.352 mg mg^−1^ ± 0.028), but no effect was noted at week six. At week six, parasitism caused an approximately 66% increase in the relative belowground biomass, while N supply had no effect. 

### 2.3. Leaflet Mass and Area were Reduced by Parasitism, while Specific Leaf Area Increased

**Figure 3:**
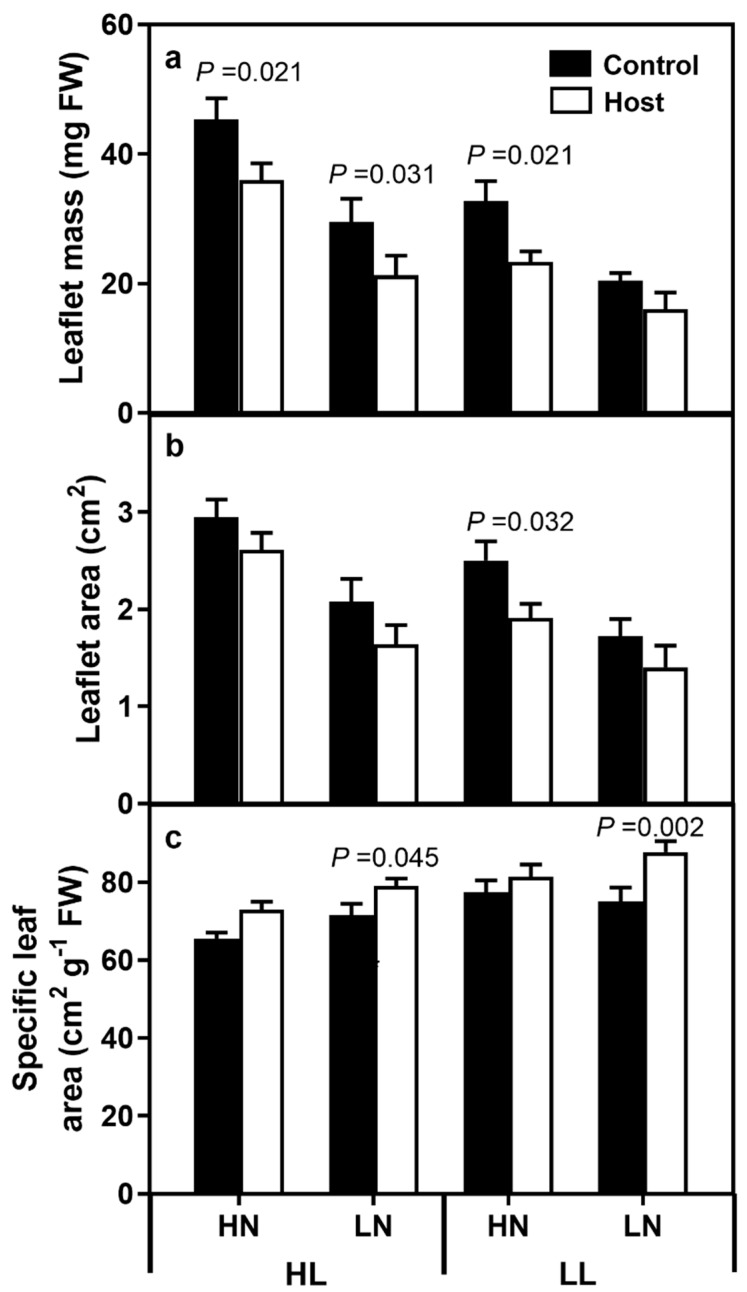
Leaflet mass (**a**), area (**b**) and specific leaf area (**c**) of clover plants, where the bars represent standard errors. *p*-values denote significant differences between control and parasitised plants. HL and LL represent high light and low light treatments, while HN and LN represent high and low N treatments.

Leaflet mass was significantly lower in plants grown under the stressed conditions (Figure 3, Table S3). The area of the host leaflets was 26.4% higher in HL than LL plants, 49.8% higher in HN plants and 19.6% higher in unparasitised plants, with no interactions noted between treatments. 

Specific leaf area (SLA; cm^2^ g^−1^ FW) was calculated by dividing the leaf area by its mass, and was 11.3% higher in LL than HL plants, 5.4% higher in LN plants, and 10.9% higher in parasitised than control plants. Significant differences between the SLA of control and parasitised plants were noted in the LN plants at both HL and LL conditions (HL–LN Control 71.6 cm^2^ g^−1^ ± 2.6, Parasitised 79.1 cm^2^ g^−1^ ± 2.5, F_(1,70)_ = 4.181, *p* = 0.045; LL–LN Control 75.2 cm^2^ g^−1^ ± 2.6, Parasitised 87.8 cm^2^ g^−1^ ± 2.8 F_(1,70)_ = 10.747, *p* = 0.002) treatments, while the difference between control and parasitised HL–HN plants was not significant (F_(1,70)_ = 3.640, *p* = 0.061). 

### 2.4. Host Photosynthesis Decreased in Response to Parasitism

**Figure 4:**
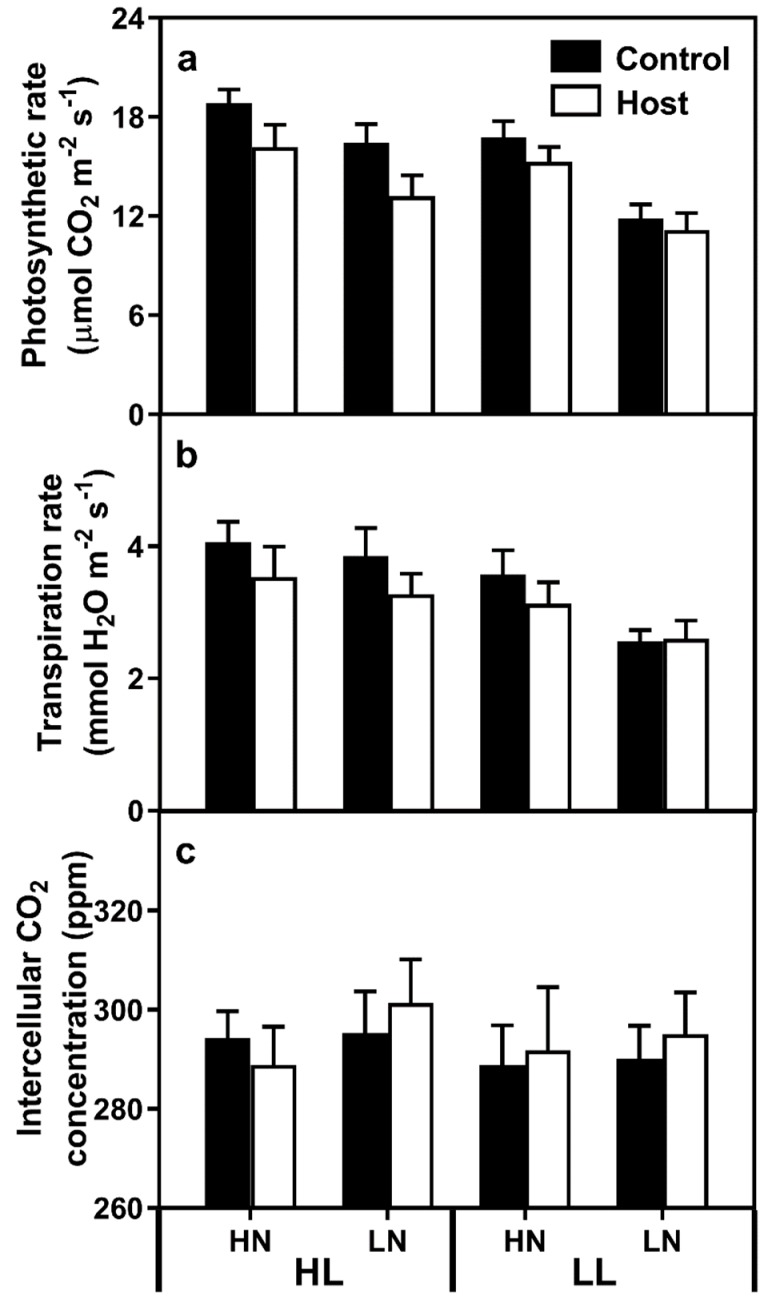
Leaf photosynthetic rate (**a**), transpiration rate (**b**) and intercellular CO_2_ concentration (**c**) of clover leaves, where the bars represent standard errors. HL and LL represent high light and low light treatments, while HN and LN represent high and low N treatments.

Expressed on a leaf area basis, light-saturated photosynthetic rates were lower in plants grown under LL, LN and parasitised conditions (Figure 4a; Table S4). No interaction of effects was noted between treatments. Similarly, on a leaf area basis, transpiration rates were 20.5% lower under LL conditions, while LN (Figure 4b; 13.1% reduction; F_(1,70)_ = 3.787, *p* = 0.056) and parasitism did not have significant effects (11.9% reduction; F_(1,70)_=3.072, *p* = 0.084). A strong correlation between photosynthetic rates and transpiration rates was noted (r = 0.814; *p* < 0.001), but no treatment effects were noted on intercellular CO_2_ levels (Figure 4c).

### 2.5. Parasitism Caused an Increase in Soluble Sugar and Chlorophyll Levels, Decreased Shoot N Levels, and had no Effect on Leaf Starch Concentration

**Figure 5:**
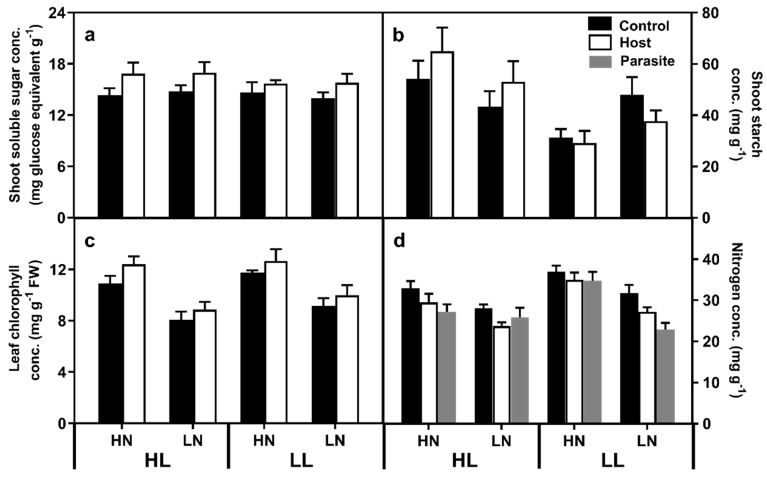
Leaf soluble sugar (**a**), starch (**b**), chlorophyll (**c**) and N concentration (**d**), where the bars represent standard errors. HL and LL represent high light and low light treatments, while HN and LN represent high and low N treatments.

Neither light nor N supply had any effect on measured leaf soluble sugar levels (Figure 5a, Table S5). However, parasitised plants exhibited leaf soluble sugar levels 13.1% higher than control plants. A light × N level effect on starch levels was noted, with LN plants exhibiting higher shoot starch concentrations than the HN plants under LL conditions (Figure 5b; F_(1,68)_ = 6.539, *p* = 0.013).

Host leaf chlorophyll levels were 32.3% higher in HN plants (Figure 5c) and 10.0% higher in parasitised than control plants. Chlorophyll levels were 8.3% higher in LL than HL plants, although this difference was not statistically significant (F_(1,69)_ = 3.342, *p* = 0.072). No effect interactions were found between any of the treatments. Host shoot N concentrations were lower in HL than LL plants (Figure 5d), under LN conditions, and in parasitised plants, with no significant effect interactions between factors found. A light x N level interaction in parasite N concentration was found, with a 51.7% increase in LL plants (HN 34.8 mg g^−1^ ± 2.2; LN 22.9 mg g^−1^ ± 1.6; F_(1,34)_ = 15.727, *p* < 0.001).

## 3. Discussion

Three weeks after the start of treatments, the HL plants had achieved a higher biomass than their LL counterparts. Given the strong relationship between host photosynthetic and growth rates [20,21], this confirms that our light treatment was sufficient to cause a significant change in plant C balance. At the three-week harvest, parasite attachment had no effect on system biomass. While the plants in all treatments grew significantly between weeks three and six, both N supply and light level exhibited a strong influence on the final biomass. LN plants were significantly smaller after six weeks of treatments, suggesting that even in the absence of the parasite, under our growth conditions the C costs of N fixation were sufficient to significantly reduce growth rates. Previous studies have demonstrated a close link between plant C status and N fixation rates in a variety of legume species, with plants grown under shaded or short photoperiod conditions fixing less N [22,23]. In our study, although we did not quantify N fixation rates, large, well developed root nodules were clearly visible, suggesting N-fixing activity. Parasitism caused a decrease in system mass, with the largest declines in the HL–HN plants, smaller declines in plants receiving either of the single stresses, and the smallest declines in the LL–LN plants. Under LL and LN conditions, the hosts were presumably already resource-depleted, limiting the parasites’ ability to further abstract resources and suppress growth. The noted lack of difference in shoot mass as a result of *O. minor* parasitism in young plants, with differences only manifesting in older red clover plants, mirrors previous studies [24]. 

The ratio of belowground biomass (roots and parasites) to shoot biomass was used to give a measure of the differences in the balance between non-photosynthetic and photosynthetic biomass. The ratio of non-photosynthetic to photosynthetic biomass diverged over time between unparasitised and parasitised plants, with this divergence both more rapid and more pronounced in the HL plants. Host root biomass per unit shoot tissue was significantly reduced in parasitised plants, but otherwise remained remarkably constant irrespective of treatment. Assuming that host root respiration rates did not significantly differ between control and parasitised plants [7], it seems likely that the additional C demands of the parasite limited host plant growth and caused the decrease in host root to shoot ratio [25]. Similarly, Hibberd et al. [5] showed that despite a large increase in C flux to the roots in parasitised tobacco plants compared to controls, resource abstraction by *O. cernua* led to a 55% decrease in C allocation to the host roots, which was associated with reduced growth rates. 

At both the three-week and six-week harvests, *O. minor* parasite mass was significantly greater in the HL plants, suggesting that parasite growth rate was primarily mediated by the host photosynthetic rate, while N supply had no direct effect on parasite mass. Previous studies have suggested that the proportion of host C allocated to *O. minor* by red clover was relatively constant, irrespective of the number of parasites or host plant size. In a greenhouse experiment, Lins et al. [24] noted that the *O. minor* parasite accounted for 35–40% of system biomass, while in our experiment, the parasite accounted for approximately 21% and 11% in the HL and LL conditions, respectively, suggesting that parasite growth rates were strongly C-limited in our experiment. The parasite constituted a greater percentage of system mass at six weeks than at week three, indicating that it was growing more rapidly than the host between the two harvest dates, and it is plausible it would have achieved similar results to those found by Lins et al. [24] if the treatments had continued for longer. In our experiment, while N deprivation was clearly sufficient to cause significant reductions in host growth rates, which we attributed to the higher C costs of N fixation compared to the assimilation of exogenously supplied N, this had no effect on parasite growth rates, which were a constant percentage of the host mass, mediated by light intensity. Despite the lack of effect of N availability on parasite growth, elevated N concentrations were noted in parasites in the LL–HN treatment. The reason for these elevated levels was not clear, but an increase in N uptake was previously noted in tobacco parasitised by *O. cernua* [7]. 

Individual leaf mass decreased under both LL and LN conditions, with parasitism causing a further decrease in leaf mass under all conditions, except the LL–LN treatment. Specific leaf area tended to increase under LL and LN conditions, which is a phenomenon typically observed in leaves growing under shade conditions and is consistent with C abstraction by the parasite [6]. Similarly, leaf chlorophyll levels tended towards being higher in low light plants, which would further increase light harvesting efficiency. Leaf chlorophyll levels were strongly decreased by the LN treatments, suggesting a higher relative N cost of chlorophyll production. In our data, leaf photosynthetic and transpiration rates were significantly decreased by both LL and LN treatments, while parasitism caused a decrease in photosynthesis but not transpiration. This contrasts with the data of Dale and Press [25], who found no difference in clover host photosynthetic rates as a result of *O. minor* parasitism, and Hibberd et al. [6], who found no difference in the photosynthetic rates of young leaves, and an increase in old leaves, in tobacco plants parasitised by *O. cernua*. In our data, given the lack of difference in calculated intercellular CO_2_ levels, changes in stomatal characteristics, implied by reduced transpiration rates, are unlikely to have caused the noted decreases in photosynthesis, suggesting another cause, such as reduced N concentrations. High light plants exhibited higher photosynthetic rates despite lower leaf N concentrations, potentially due to changes in leaf architecture. Pincovici et al. [8] similarly found a decline in light-saturated photosynthetic levels on an area basis, although the differences were mitigated when expressed on a “per leaf” basis, due to increased leaf areas in parasitised plants. In our experiment, leaflet areas were smaller in parasitised than control plants, which would exacerbate photosynthetic declines in parasitised plants. 

N fixation is energetically expensive [9], and under light-limited conditions, might be expected to limit growth in N-limited plants. Indeed, this is clearly visible in our data, where the HN plants grew to a greater extent than their LN counterparts. Interestingly, N deficit and light limitation had broadly similar impacts on biomass accumulation between the two harvest dates. A core expectation of our experiment was that we would find an N-level x parasitism interaction, such that LN plants would be more strongly affected by parasitism than HN plants. While parasitism caused a significant decrease in host shoot N concentration, no N level x parasitism interaction was found, and the total mass of LN systems were less affected by parasitism than HN systems. Similarly, exogenous N supply had no effect on parasite growth, suggesting that the posited competition for C between the parasite and N fixation was of minor importance in our plants. Conversely, host shoot growth declined both as a result of N deficiency and parasitism, which suggests C competition between shoot growth and both the root nodules and the parasite. 

We hypothesised that leaf sugar levels would decrease as a result of C abstraction by the host. However, our soluble sugar data showed the opposite trend, with parasitised plants exhibiting significantly higher concentrations than controls. The reason for this is not clear, but similar increases in soluble sugar levels have also been found in tomato plants parasitised by *O. cernua* [26]. It is plausible that these elevated soluble sugar concentrations are a result of reduced sugar loading into the phloem, or a strategy to shift the plants’ osmotic balance towards the leaves. Starch levels were lower in the LL plants, and particularly in the LL–HN plants, which also tended to show high tissue N concentrations, suggesting that host–leaf starch accumulation may have been suppressed by the C-costs of N uptake and assimilation. However, no main effect of parasitism on starch levels was noted [25], although a light x parasitism interaction approached significance, suggesting that any effects of parasitism may be contingent on photosynthetic rates. 

## 4. Materials and Methods 

*Orobanche minor* L. seeds were collected from a natural population at the University of Tsukuba campus in May 2017. *Trifolium pratense* Sm. seeds were purchased from a commercial seller (Takii seeds, Kyoto, Japan). 10.5 cm diameter pots with a volume of 0.5 L were half filled with vermiculite, and 5 mg per pot of *O. minor* seeds were spread in half the pots. The pots were filled to 80% and 50 mg of a commercial rhizobium culture was spread in each pot (Mame-zou, Tokachi Agricultural Cooperative Association, Obihiro, Japan). Thus, the layer of *O. minor* seeds was approximately 4 cm from the top of the pot, while the rhizobia layer was approximately 2 cm from the surface of the vermiculite. The pots were filled, moistened with deionised water and three *T. pratense* seeds were sown at 5 mm depth in each pot. Seed colour was not considered at sowing. After a week the seedlings were thinned to one plant per pot. Plants were grown under control conditions for five weeks to establish themselves, and were supplied 20 mL of a full-strength Broughton and Dilworth nutrient solution [27] supplemented with 5 mM ammonium nitrate three times a week. Broughton and Dilworth solution is an N-free nutrient solution formulated specifically for legumes. A previous study [25] reported that parasite attachment occurred around 30 days after planting, so for three weeks, from day 14 through day 35, plants were provided a nutrient solution with P supplied at 10% of a full strength solution, to promote strigolactone production and parasite germination and attachment [19]. Plants were grown at 24 °C under fluorescent lights providing approximately 180 μmol photons m^−2^ s^−1^, with a 14-h photoperiod. 

From week five onwards, half the plants were transferred to low light (LL) conditions (80 μmol photons m^−2^ s^−1^), while half remained at the higher light intensity (HL). Plants received the same nutrient solution, but half the plants at each light level received no exogenous N supply (LN), while half received the supplementary 5 mM ammonium nitrate (HN). This yielded a 2 (light) × 2 (N) × 2 (parasite vs control) fully factorial design with a minimum of 18 replicates. The aim of the LL and LN treatments was to impose C and N deficits, respectively.

The experimental treatments were imposed for six weeks, with a midpoint harvest after three weeks. At the three-week point, six replicate plants from each treatment were harvested, separated into shoots, roots and parasites (where applicable), oven dried and weighed. Two days prior to the final harvest (i.e., 40 days after the start of the treatments), 10 replicate plants (plus replacements for unparasitised plants) from each treatment had the photosynthetic and transpiration rates of the centre leaflet from a randomly selected leaf measured using a LiCor LI6400XP providing 1000 μmol photons m^−2^ s^−1^, at 400 ppm CO_2_, with a leaf temperature of 25 °C. Along with the photosynthetic and transpiration rates, the calculated intercellular CO_2_ concentration was downloaded from the LI6400XP. The measured leaflet was detached from the plant, weighed and scanned (Canon LiDE 220), and its area measured using ImageJ. The specific leaf area was calculated by dividing the leaf area by the measured mass. The leaflet was imbibed in methanol containing 10 mg L^−1^ MgSO_4_ for a minimum of 36 h before the chlorophyll was quantified photometrically [28]. Six weeks from the start of the treatment, plants which had their photosynthetic rates measured were harvested and dried as previously stated. Where parasites did not attach, the plants were replaced where possible, yielding 9 to 11 replicates per treatment. After weighing, the shoots were homogenised into 1 mm segments and 10 mg subsamples were digested by micro-Kjeldahl method, and their N concentration determined by Nessler’s reagent [29].

Alcohol soluble carbohydrate and starch contents were quantified by the method of Chow and Landhäusser [30]. Briefly, soluble sugars were extracted from 20 mg of dried host shoot material by heating in a test tube for 10 min at 95 °C in 5 mL of 80% ethanol. 0.5 mL of extract was transferred into two tubes. To one tube, 1 ml of a 2% phenol solution was added, while to the second tube 1 mL of deionised water was added as a control. 2.5 mL of concentrated sulphuric acid was added to both tubes and the colour allowed to develop for 10 min, at which point the tubes were transferred to a water bath at room temperature to cool. After 30 min, the absorbance was read at 490 nm against glucose standards prepared in 80% ethanol.

Starch was quantified on a second tissue sample. Soluble sugars and chlorophyll were extracted three times at 95 °C in 80% ethanol. After the supernatant was discarded, starch was extracted from the remaining pellet by heating with 2 mL of 0.1 M NaOH at 50 °C for 30 min. The sample was neutralised by the addition of 2.5 mL of 0.1 M acetic acid, 0.5 mL of digest buffer was added, and the sample incubated at 50 °C for 24 h before quantification. The digest buffer was a 0.05 M acetate buffer (pH 5.1), containing 1000 U mL^−1^ α-amylase and 6 U ml^−1^ amyloglucosidase (Sigma-Aldrich). Sugars were quantified from 0.2 mL aliquots of the sample by the addition of 1.8 mL of PGO solution. The PGO solution was made by dissolving 1 capsule of PGO enzymes (Sigma P-7119) in 100 mL of water containing 1.6 mL of *o*-dianisidine solution (50 mg of *o*-dianisidine dihydrochloride in 20 mL of deionised water). After allowing the colour to develop for 30 min in the dark, the absorbance was measured at 450 nm against suitable starch standards.

Statistical analyses were conducted in IBM SPSS Statistics v25.0. Differences between means were analysed by factorial ANOVA, using type III sum-of-squares, which accounts for the unbalanced design. Where significant interactions between effects were noted, simple effects analysis with a Šidák correction was used. Where the data failed homogeneity of variance testing (Levene’s test), it was log transformed before analysis, which achieved homoscedasticity in the vast majority of cases. Correlation analysis was conducted using the Pearson correlation command. In the text, we report statistical analyses reporting *p*-values of 0.05 or below as statistically significant. Where the *p*-values were between 0.05 and 0.1, we reported them in the text. 

## 5. Conclusions

We hypothesised that under low light, N-deficient conditions, we would find evidence of C competition between N fixation and parasite growth. Contrary to our expectations, infection by *O. minor* had little effect on clover host plant biomass under ostensibly C-limited conditions, with parasitism causing the largest decrease in host biomass under high light conditions where N was supplied. Parasite mass was directly related to light levels and represented additional belowground biomass, beyond host root mass, which scaled isometrically with shoot mass. N supply had no effect on parasite mass, which appeared to be a relatively constant proportion of system mass at each light level. We hypothesised that parasitism would cause significant decreases in plant C status. However, our data revealed a small increase in host leaf soluble sugar concentrations, with parasite attachment having no effect on starch levels. No evidence of competition between the root nodules and the parasites for C was noted, although both N fixation and parasitism caused declines in host growth rate.

## Supplementary Materials

The following are available online at https://www.mdpi.com/2223-7747/8/6/146/s1, Table S1: Statistical analysis of log-transformed biomass data of parasitised and control clover plant biomass three and six weeks after the start of light and N treatments. Table S2: Belowground mass per unit shoot mass (mg mg^−1^) of control or *O. minor*-infected *Trifolium pratense* plants grown under high and low light levels, and either supplied or deprived of exogenous nitrogen. Table S3: Results of a 3-way ANOVA of clover host leaf physical properties in the presence or absence of parasitism by *O. minor*, Table S4: Results of a 3-way ANOVA of clover host leaf photosynthetic properties in the presence or absence of parasitism by *O. minor.* Table S5: Results of a Factorial ANOVA of clover host leaf biochemical properties in the presence or absence of parasitism by *O. minor*.
