# Supplementary material for: Effects of Light Level and Nitrogen Supply on the Red Clover–Orobanche Minor Host–Parasite Interaction
Source: Plants (Basel). 2019 May 31;8(6):146. doi: 10.3390/plants8060146 (PMC6631064; doi:10.3390/plants8060146)
Supplement: Supplementary file 1 [file plants-08-00146-s001.pdf]

Supplementary table 1; Statistical analysis of log-transformed biomass data of parasitized and control clover plant biomass three and six weeks after the start of light and N treatments. The Week 3 Shoot data returned a Levene's test value of 0.041 and should be interpreted with caution. Asterisks denote statistical significance as P<0.05 (*), <0.01 (**) and <0.001 (***).

		Week 3		Week 6	
		df	F-value	df	F-value
System	Light level	1	13.109***	1	31.330***
	N level	1	1.043	1	26.629***
	Parasitism	1	1.311	1	4.609*
	Light x N level	1	0.013	1	2.537
	Light x Parasitism	1	0.012	1	0.472
	N level x Parasitism	1	0.014	1	0.345
	Light x N level x Parasitism	1	0.240	1	0.212
	Error	37		70	
Shoot	Light level	1	10.736**	1	27.036***
	N level	1	0.169	1	29.819***
	Parasitism	1	2.913	1	13.903***
	Light x N level	1	0.075	1	1.527
	Light x Parasitism	1	0.094	1	1.265
	N level x Parasitism	1	<0.001	1	0.581
	Light x N level x Parasitism	1	0.199	1	0.206
	Error	37		70	
Belowground	Light level	1	12.385**	1	33.483***
	N level	1	4.233*	1	17.141***
	Parasitism	1	0.003	1	0.270
	Light x N level	1	0.117	1	4.329
	Light x Parasitism	1	0.599	1	0.013
	N level x Parasitism	1	0.048	1	0.046
	Light x N level x Parasitism	1	0.091	1	0.336
	Error	37		70	

Supplementary table 2; Belowground mass per unit shoot mass (mg mg^{-1}) of control or *O. minor*-infected *Trifolium pratense* plants grown under high and low light levels, and either supplied or deprived of exogenous nitrogen.

		Week 3		Week 6	
		d.f.	F-value	d.f.	F-value
Host root only	Light	1	0.204	1	0.276
	N level	1	6.651*	1	0.134
	Parasitism	1	4.246*	1	4.653*
	Light x N level	1	0.150	1	4.994*
	Light x Parasitism	1	0.005	1	0.217
	N level x Parasitism	1	0.004	1	2.861
	Light x N level x Parasitism	1	0.508	1	0.645
	Error	37		70	
Parasite only	Light	1	7.949*	1	6.517*
	N level	1	2.751	1	0.092
	Light x N level	1	1.643	1	0.330
	Error	17		34	
Host root + parasite	Light	1	4.626*	1	8.430**
	N level	1	6.502*	1	0.872
	Parasitism	1	4.272*	1	56.545***
	Light x N level	1	0.281	1	4.583*
	Light x Parasitism	1	3.000	1	2.918
	N level x Parasitism	1	0.218	1	0.768
	Light x N level x Parasitism	1	0.126	1	0.165
	Error	37		70	

Supplementary table 3; Results of a 3-way ANOVA of clover host leaf physical properties in the presence or absence of parasitism by *O. minor*.

		F-values		
	d.f.	Mass	Area	SLA
Light	1	25.668***	13.180**	18.501***
N level	1	41.125***	38.566***	4.497*
Parasitism	1	16.113***	7.756**	17.104***
Light x N level	1	1.915	0.450	1.228
Light x Parasitism	1	0.250	0.012	0.056
N level x Parasitism	1	0.603	0.466	1.324
Light x N level x Parasitism	1	0.248	1.154	1.240
Error	70			

Supplementary table 4; Results of a 3-way ANOVA of clover host leaf photosynthetic properties in the presence or absence of parasitism by *O. minor*

		F-values		
	d.f.	PS	Transpiration	Ci
Light	1	10.607**	10.094**	1.376
N level	1	22.053***	3.787	1.841
Parasitism	1	7.568**	3.072	0.032
Light x N level	1	1.280	0.891	0.029
Light x Parasitism	1	1.136	0.312	0.063
N level x Parasitism	1	0.018	0.405	1.320
Light x N level x Parasitism	1	0.261	0.535	0.022
Error	70			

Supplementary table 5; Results of a Factorial ANOVA of clover host leaf biochemical properties in the presence or absence of parasitism by *O. minor*. Soluble sugars data was log transformed before analysis.

		d.f.	F-value
Soluble sugars	Light	1	0.740
	N level	1	<0.001
	Parasitism	1	6.919**
	Light x N level	1	0.162
	Light x Parasitism	1	0.106
	N level x Parasitism	1	0.019
	Light x N level x Parasitism	1	0.116
	Error	70	
Starch	Light	1	16.214***
	N level	1	0.506
	Parasitism	1	0.024
	Light x N level	1	8.717**
	Light x Parasitism	1	3.124
	N level x Parasitism	1	0.015
	Light x N level x Parasitism	1	0.051
	Error	68	
Chlorophyll	Light	1	3.342
	N level	1	40.793***
	Parasitism	1	4.798*
	Light x N level	1	0.391
	Light x Parasitism	1	0.091
	N level x Parasitism	1	0.176
	Light x N level x Parasitism	1	0.123
	Error	69	
Host N concentration	Light	1	14.431***
	N level	1	29.046***
	Parasitism	1	10.860***
	Light x N level	1	0.280
	Light x Parasitism	1	0.089
	N level x Parasitism	1	0.627
	Light x N level x Parasitism	1	0.155
	Error	70	
Parasite N concentration	Light	1	1.285
	N level	1	10.324**
	Light x N level	34	6.387*
